# A Conserved Domain in the Scc3 Subunit of Cohesin Mediates the Interaction with Both Mcd1 and the Cohesin Loader Complex

**DOI:** 10.1371/journal.pgen.1005036

**Published:** 2015-03-06

**Authors:** Ola Orgil, Avi Matityahu, Thomas Eng, Vincent Guacci, Douglas Koshland, Itay Onn

**Affiliations:** 1 Faculty of Medicine in The Galilee, Bar-Ilan University, Safed, Israel; 2 Department of Molecular and Cell Biology, University of California, Berkeley, Berkeley, California, United States of America; University of Dresden, Germany

## Abstract

The Structural Maintenance of Chromosome (SMC) complex, termed cohesin, is essential for sister chromatid cohesion. Cohesin is also important for chromosome condensation, DNA repair, and gene expression. Cohesin is comprised of Scc3, Mcd1, Smc1, and Smc3. Scc3 also binds Pds5 and Wpl1, cohesin-associated proteins that regulate cohesin function, and to the Scc2/4 cohesin loader. We mutagenized *SCC3* to elucidate its role in cohesin function. A 5 amino acid insertion after Scc3 residue I358, or a missense mutation of residue D373 in the adjacent stromalin conservative domain (SCD) induce inviability and defects in both cohesion and cohesin binding to chromosomes. The I358 and D373 mutants abrogate Scc3 binding to Mcd1. These results define an Scc3 region extending from I358 through the SCD required for binding Mcd1, cohesin localization to chromosomes and cohesion. Scc3 binding to the cohesin loader, Pds5 and Wpl1 are unaffected in I358 mutant and the loader still binds the cohesin core trimer (Mcd1, Smc1 and Smc3). Thus, Scc3 plays a critical role in cohesin binding to chromosomes and cohesion at a step distinct from loader binding to the cohesin trimer. We show that residues Y371 and K372 within the SCD are critical for viability and chromosome condensation but dispensable for cohesion. However, scc3 Y371A and scc3 K372A bind normally to Mcd1. These alleles also provide evidence that Scc3 has distinct mechanisms of cohesin loading to different loci. The cohesion-competence, condensation-incompetence of Y371 and K372 mutants suggests that cohesin has at least one activity required specifically for condensation.

## Author Summary

Cohesin is a four-subunit complex that tethers distinct regions of chromatin either intra- or inter-molecularly. Cohesin is essential for mediating proper chromosome structure and dynamics and to maintain genome stability. In humans, mutations in cohesin are associated with several developmental disorders and cancer. The function of the Scc3 subunit of cohesin is unclear. In this study we use a genetic approach to isolate mutations in *SCC3* that abrogate cohesin function. We identified a mutant adjacent to an evolutionarily conserved domain of Scc3 termed the SCD. This mutant retained the ability to interact with the cohesin loader and the cohesin regulatory proteins Wpl1 and Pds5. However, it abrogated the interaction with the cohesin core trimer (Mcd1, Smc1, Smc3). This interaction was found to be critical for the initial association of cohesin with chromosomes and for its tethering activity. In-depth analyses of the SCD revealed a subset of mutants that retained their ability to interact with the cohesin trimer and had normal cohesion but were inviable and defective for condensation. These results reveal that the SCD region of Scc3 plays a role in chromosome condensation distinct from that in cohesion. Our results shed a new light on the mechanisms whereby cohesin helps maintains genome stability.

## Introduction

Higher order chromosome structure is essential for promoting genome integrity [1,2]. The cohesin protein complex mediates chromosome structure by tethering distal chromosomal regions either inter- or intra-molecularly [3–7]. Cohesin tethers the newly replicated DNA molecules, known as sister chromatids, from their formation during S phase until their separation during anaphase of mitosis or meiosis II. Cohesin ensures bipolar attachment of chromatids to the spindle, as well as proper chromosome condensation, both of which are molecular safeguards against genome instability [1,3,8]. In addition, cohesin regulates gene expression and is involved in DNA repair [9–13]. The multi-functionality of cohesin requires sophisticated regulation. In humans, mutations in cohesin and its regulators are associated with several developmental disorders and cancer, making the study of cohesin medically relevant [10,14–16].

**Fig 1:**
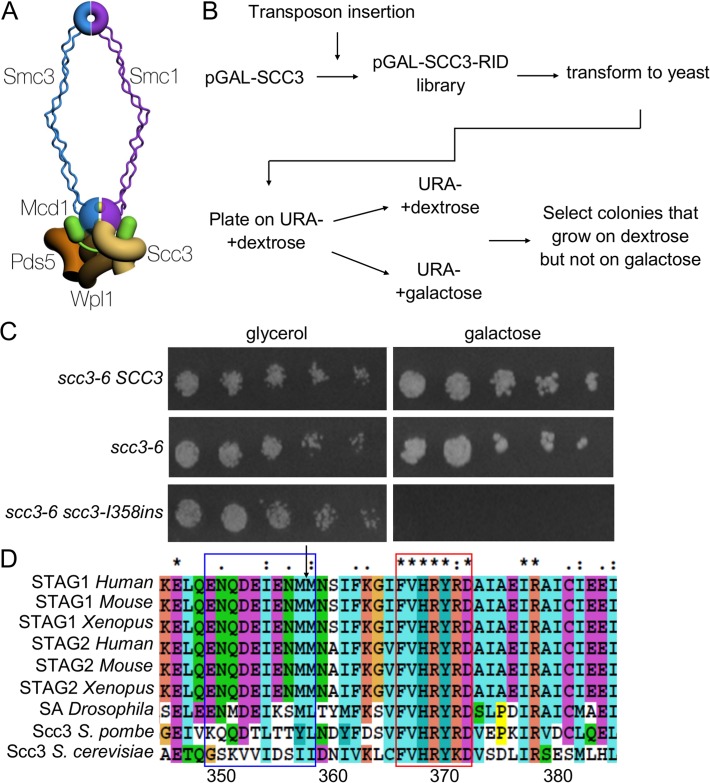
Identification of a functional domain in Scc3. **A.** Schematic of the cohesin complex. The two yellow balls between the Smc1 and Smc3 represent ATP molecules. **B.** Flowchart of the experimental design to identify *SCC3* dominant negative mutants. **C.** Strain VG3135 (*scc3-6*) cells carrying pRS406 (*pGALURA3*), pIO88 (*pGAL*-*SCC3 URA3*) or pIO88R1 (*pGAL*-*scc3-I358insURA3*) were grown to saturation in SC-URA media. Fivefold serial dilutions of each strain was plated on SC-URA plates containing either glycerol or galactose and grown at 23°C. **D.** Sequence alignment of Scc3 was generated by ClustalX. The arrow indicates the insertion site after I358. The blue and red frames indicate the RID A region G350-I359 and the conserved sequence F367-D373, respectively.

Scc3 (also referred to as Irr1/Stag/SA1/2) is one of four conserved subunits of cohesin. The other subunits include two proteins of the Structural Maintenance of Chromosomes (SMC) family called Smc1 and Smc3, and another non-Smc protein called Mcd1/Scc1/Rad21 (Fig. 1A). The Smc1-Smc3-Mcd1 subunits and their contribution to cohesin function and regulation have been studied extensively [1,2]. In contrast, Scc3 has been the primary subject of only a few studies leaving its function a major question in the field and the focus of this work.

The few studies of Scc3 suggest that it plays a complex role in cohesin structure and function. The *SCC3* gene is essential for cell viability. The initial *scc3* temperature sensitive (ts) allele (*scc3-1*) described in budding yeast induced a major cohesion defect [17]. Depletion or mutation of Scc3 in other organisms also induces a cohesion defect [18–20]. These studies indicate that the Scc3 function in cohesion is evolutionarily conserved. In the *scc3-1* mutant, the cohesin trimer composed of Mcd1, Smc1 and Smc3 remains intact [21,22]. However, the trimer fails to localize to chromosomes, indicating either its inability to bind chromosomes or to remain stably bound [17,23,24]. Evidence for both possibilities exists. Cohesin is loaded onto chromosomes by an Scc2/Scc4 loader complex [25]. Recent work has identified an interaction between the loader and Scc3 that is important for cohesin’s ability to bind DNA *in vitro*, and for generating cohesion *in vivo* [26]. Scc3 is also part of a second complex that contains two other conserved cohesin regulators, Wpl1 and Pds5. Wpl1 is a cohesion inhibitor thought to destabilize cohesin binding to chromosomes and Pds5 is a cohesion maintenance factor [23,24,27,28]. Thus it is possible that scc3 mutants might destabilize cohesin binding to chromosomes by activating Wpl1 or failing to inhibit it.

The ability of Scc3 to interact with both cohesin and its auxiliary factors raises important questions regarding its role in cohesin function. For example, *in vitro* experiments suggest that an evolutionarily conserved ∼90 amino acid SCC3 domain called the stromalin conservative domain (SCD; a.a. 367–457, Interpro accession #: IPR020839) binds Mcd1 to enable Scc3 interaction with the cohesin trimer [29,30]. *In vitro* peptide binding analyses revealed that the cohesin loader Scc2 subunit interacts with Scc3, as well as with each member of the cohesin trimer [26,31]. These observations raise several questions. How important is the Scc3-Mcd1 interaction for the recruitment of Scc2 to the cohesin trimer? Does the Scc3-Mcd1 interaction contribute to cohesion, or alternatively, is the Scc3-Scc2 interaction sufficient for cohesion? Does the Scc3-Scc2 interaction depend upon Scc3's interaction with any of its other binding partners, such as Mcd1, Pds5 or Wpl1? Clearly, understanding the molecular functions of Scc3 in cohesion will require mutations in Scc3 that disrupt individual interactions among its multiple binding partners.

Recent studies raise the prospect that Scc3 has an additional role in chromosome structure, which is hinted by phenotypic similarities in mutants of the Scc3, Wpl1 and Pds5 trimer. Specific mutations in *SCC3* and *PDS5* or null mutations in *WPL1* (*wpl1*Δ) overcome the normal requirement for the Eco1 acetyltransferase for cell viability [23,24]. This restoration of viability was assumed to result from the restoration of cohesion establishment because Eco1-dependent acetylation of Smc3 during S phase is known to be critical for this step [32,33]. However, cells containing both an *eco1* defective allele and suppressor mutations in *SCC3*, *WPL1* or *PDS5* were, surprisingly, as defective for cohesion as the *eco1* parent or even cohesin subunits mutants [23,24,34]. Additional analyses suggest that yeast can tolerate a severe cohesion defect because they have an alternative cohesin-independent pathway for bipolar attachment of sister chromatids [35]. Interestingly, *eco1* cells are also defective for condensation, which is suppressed by *wpl1*Δ [35]. This correlation between suppression of condensation by *wpl1*Δ and restoration of viability suggests that the condensation defect in *eco1* cells is the cause of their inviability, and that Wpl1 is a condensation inhibitor. Thus, the previously reported restoration of viability of *eco1*Δ cells by certain *scc3* mutations may also reflect a heretofore unappreciated role of Scc3 in regulating chromosome condensation.

In this study we used a genetic approach to identify functional domains in budding yeast Scc3. We find that mutations in a short region of the SCD encompassing residues 358 to 373 blocked Scc3 binding to Mcd1. Exploiting these alleles, we show that the Mcd1-Scc3 interaction is necessary for both binding of cohesin to chromosomes and for cohesion, but not for the recruitment of Scc2 to either Scc3 or cohesin. Thus, the Mcd1-Scc3 interface is critical for loading at a step distinct from Scc2 recruitment to cohesin. Finally, we show that residues within the SCD domain are specifically required for condensation but not cohesion, revealing a novel activity of Scc3 as a critical regulator of condensation.

## Results

### A genetic screen to identify functional domains in Scc3

A mutagenesis strategy called Random Insertion of Dominant (RID) was used to identify functional domains in Scc3 [36]. A library of Scc3 RID mutants was constructed by random insertion of a 15 bp DNA sequence into a *CEN URA3* plasmid containing the *SCC3* gene under the control of the GAL promoter (Materials and Methods). The library was transformed into haploid strain VG3135 (*scc3-6*) and assayed at the permissive temperature (23°C). We identified fourteen colonies that exhibited toxicity or lethality on galactose but grew well on glycerol, as candidate dominant Scc3-RID mutants (Fig. 1B and C, Materials and Methods). Candidate plasmids were isolated from yeast, sequenced and retransformed to confirm the overexpression-induced lethality. These fourteen RID mutations clustered into four distinct regions of Scc3. Eight of the 14 RID insertions were located between amino acids 350 and 359, henceforth referred to as the RID A region (Supplemental S3 Table). The amino acid sequence of the RID A region is not conserved but is immediately adjacent to the N terminal end of the conserved SCD (Fig. 1D). The large number of insertions in RID A, coupled with their proximity to the SCD, made them particularly interesting for additional study.

### scc3-I358ins identifies a region of Scc3 important for cohesin loading onto chromosomes and sister chromatid cohesion

**Fig 2:**
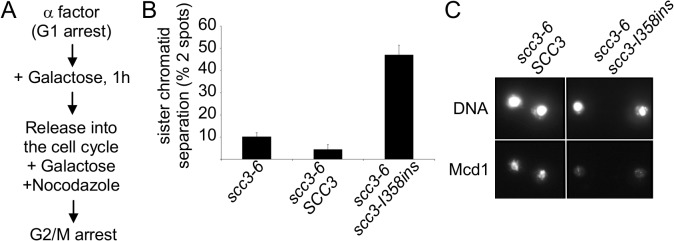
Analysis of sister chromatid cohesion upon scc3-I358ins overexpression. **A.** Flowchart of the experimental design. **B.** Strain YIO81 with plasmid pRS406 (*pGAL URA3*), pIO88 (*pGAL*-*SCC3 URA3*) or pIO88R1 (*pGAL*-*scc3*-*I358ins URA3*) were grown in YEP lactic acid (2%) to mid-log phase and arrested in G1 using α-factor. Galactose was added to a final concentration of 2% for 1 h; cells were then released into the cell cycle and rearrested in G2/M by nocodazole. Sister chromatid cohesion was analyzed by using the GFP spot assay (n = 3). **C.** Strains analyzed in B were processed for chromatin spreads after cells were arrested in G2/M, as described in the materials and methods section. Cohesin was detected by indirect immunofluorescence with an anti-Mcd1 antibody (n = 2).

To assess the molecular defect of RID A insertions, we chose to study the insertion after I358 residue of Scc3 (*scc3-I358ins*). Given cohesin’s important role in sister chromatid cohesion, we first tested whether *scc3-I358ins* overexpression induced a cohesion defect. Cultures of haploid strain YIO81 (*scc3-6*) containing the empty *pGAL* vector, *pGAL-SCC3* or *pGAL-scc3-I358ins* were arrested in G1 at 23°C using alpha factor. Galactose was added and cells incubated for 1 hour to induce the *GAL* promoter. Cells were released from G1 at 23°C into YPGAL media containing nocodazole to allow cell-cycle progression under inducing conditions through arrest in G2/M (Fig. 2A). Cohesion was analyzed at the *LYS4* locus using the LacI-GFP spot assay (Materials and Methods). In cells carrying either the empty vector or the wild-type *SCC3* allele, only about 10% of cells had two GFP spots, indicating robust cohesion at the permissive temperature of 23°C. In contrast, close to 50% of scc3-I358ins expressing cells had 2-GFP signals, indicative of precocious sister chromatid separation (Fig. 2B). Thus, over-expression of *scc3-I358ins* from G1 to G2/M induced a major cohesion defect.

Next, we explored the mechanism by which *scc3-I358ins* overexpression impaired cohesion. We first assessed whether cohesin was loaded onto chromatin. Cultures of *scc3-6* strains bearing either *pGAL-SCC3* or *pGAL-scc3-I358ins* were arrested in G1 at 23°C, galactose added to induce *SCC3* or *scc3-I358ins* expression, then released from G1 and arrested in G2/M at 23°C under inducing conditions as described in Fig. 2A. Chromosome spreads were prepared from G2/M arrested cells, and cohesin binding to chromatin assayed by immunofluorescence using an antibody against Mcd1.

Previous studies have shown that the presence of cohesin on chromosomes depends on Mcd1 and the integrity of the complex, making it a marker for cohesin binding [17]. As expected, cells overexpressing Scc3 exhibited a bright Mcd1 signal over the entire DNA mass (Fig. 2C). In contrast, cells over-expressing *scc3-I358ins* had only a faint Mcd1 signal. This dramatic reduction in the amount of Mcd1 bound to chromosomes suggested that *scc3-I358ins* over-expression interferes with either the loading step of cohesin or induced its dissociation from chromosomes.

**Fig 3:**
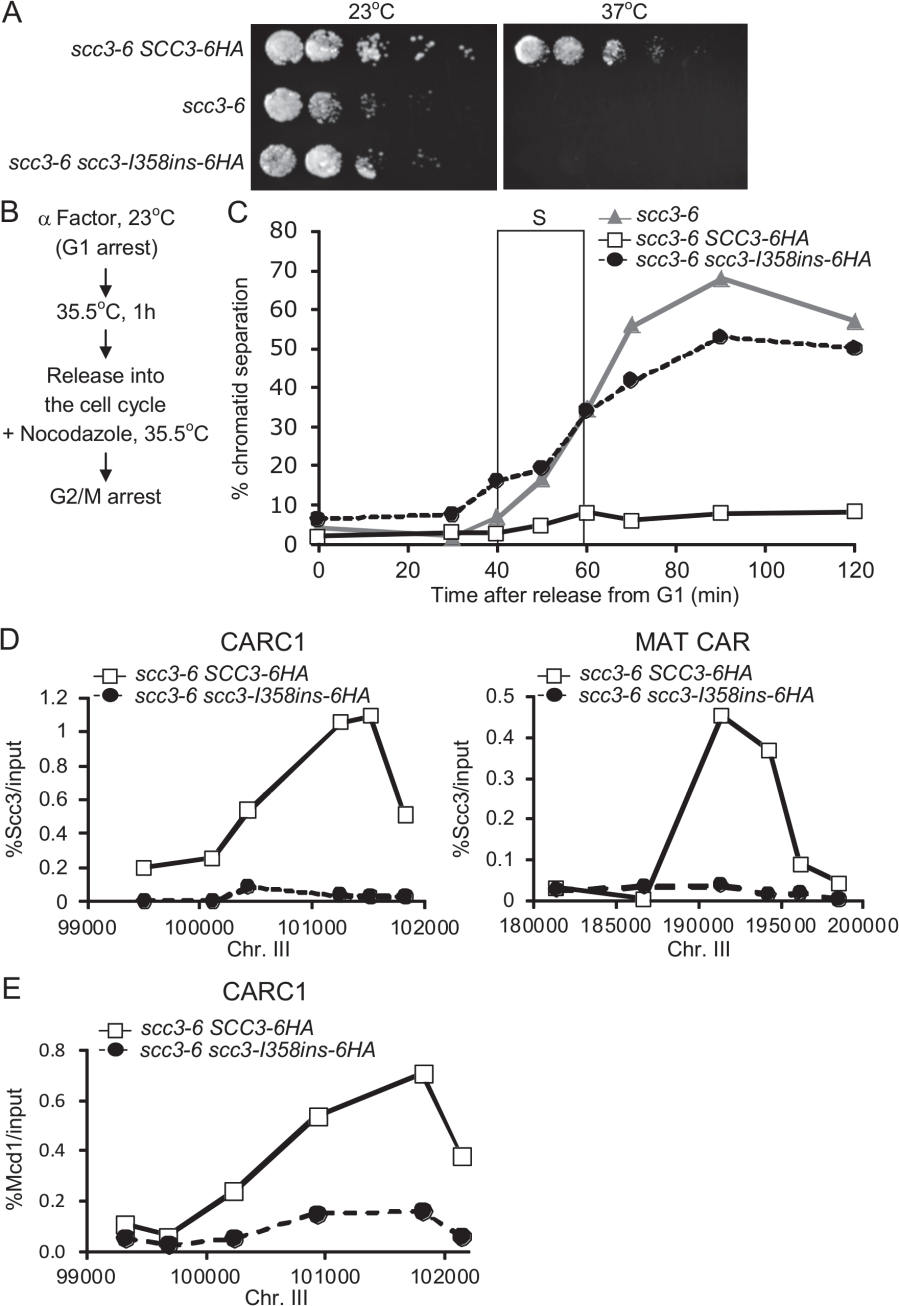
Cohesion and chromosome binding of scc3-I358Ins under native expression levels. **A.** Strains YIO81 (*scc3-6*), YIO91 (*SCC3-6HA scc3-6*) andYIO91R1 (*scc3-I358ins-6HA scc3-6*) were grown to saturation in YPD media. Fivefold serial dilutions of each strain was plated on YEPD plates and grown at either the permissive (23°C) or restrictive (37°C) temperature for *scc3-6*. **B**. Flowchart of the experimental design to score sister chromatid cohesion. **C.** Strains as indicated in A were grown at 23°C to mid-log phase and arrested in G1 using α-factor. Cells were then shifted to 35.5°C for 1 h, released into the cell cycle and re-arrested in G2/M with nocodazole. Samples for the cohesion assay were taken approximately every 15 minutes (n = 3). The frame indicates S-phase of the cell cycle as determined by flow cytometry (S1 Fig). **D.** Strains described in A were processed as in B for chromatin immunoprecipitation analysis. Scc3, HA tagged proteins were immunoprecipitated. Precipitated DNA was analyzed by quantitative PCR with six primer pairs for the MAT CAR and CARC1, as described (Material and methods). A representative experiment is shown (n = 3). **E.** Strains described in A were processed as in B for chromatin immunoprecipitation analysis. Mcd1 was immunoprecipitated. Precipitated DNA was analyzed by quantitative PCR for CARC1. A representative experiment is shown (n = 3).

To further dissect the molecular defect of scc3-I358ins, we generated *SCC3-6HA* and *scc3-I358ins-6HA* alleles, which contain six hemagglutinin epitopes (6HA) at the C-terminus, and are under control of the endogenous *SCC3* promoter. These alleles were integrated into haploid strain YIO81 (*scc3-6*) at the *URA3* locus. The YIO81 parent alone or bearing either *SCC3-6HA* or *scc3-I358ins-6HA* were grown to saturation, serially diluted on YPD plates and incubated at 23°C or 37°C, the permissive or restrictive temperature of the *scc3-6* allele, respectively (Fig. 3A). At 23°C, the *scc3-6* strain alone and the *scc3-I358ins-6HA scc3-6* strain grew equally well, but slightly slower than *SCC3-6HA scc3-6* strain. Thus the *scc3-6* allele is slightly compromised for Scc3 function at the permissive temperature, but even so, endogenous expression levels of *scc3-I358ins-6HA* is recessive in contrast to its dominant-negative phenotype when overexpressed (Figs. 1C and 3A). At 37°C, the *scc3-6* strain alone and the *scc3-I358ins-6HA scc3-6* strain fail to grow whereas the *SCC3-6HA scc3-6* strain is viable.

We interpret this expression-dependent change in phenotype as follows. The scc3-I358ins protein assembles into cohesin but is defective for one or more essential Scc3 functions. Endogenous expression levels at 23°C result in cells containing both functional scc3-6 cohesin and defective scc3-I358ins cohesin, but sufficient amounts of the former enables viability. At 37°C, scc3-6 is inactivated and the defective scc3-I358ins cohesin cannot support viability. When *scc3-I358ins* is overexpressed at 23°C, excess scc3-I358ins outcompetes scc3-6 for assembly into cohesin, rendering most or all cohesin in cells defective.

This model predicts that *scc3-I358ins-6HA scc3-6* strain at the restrictive temperature will mimic the cohesion and chromosome binding phenotypes seen with *scc3-I358ins* overexpression. To test this prediction, we first studied the kinetics of sister chromatid cohesion loss. Cells were arrested in G1 phase, shifted to the non-permissive temperature (35.5°C) for *scc3-6*, then released from G1 into media containing nocodazole at 35.5°C to re-arrest cells in G2/M (Figs. 3B and S1). Most *SCC3-6HA scc3-6* cells had a single GFP spot at all-time points, indicative of robust cohesion (Fig. 3C). In contrast, in *scc3-6* cells or in *scc3-I358ins-6HA scc3-6* cells, two GFP spots were detected concomitant with S phase, indicative of a severe defect in cohesion establishment.

We next examined whether cohesin binding to chromosomes is impaired when *scc3-I358ins-6HA* is under the endogenous *SCC3* promoter as it was when overexpressed. For this purpose we performed chromatin immunoprecipitation (ChIP) on *SCC3-6HA scc3-6* and *scc3-I358ins-6HA scc3-6* strains at non-permissive temperature. Cells were arrested in G1 phase at 23°C, shifted to 35.5°C to inactivate *scc3-6*, then released from G1 at 35.5°C and re-arrested in G2/M (Fig. 3B). G2/M arrested cells were processed for ChIP using anti-HA antibodies. Then, Scc3 binding to two known cohesin associated regions (CARs) on chromosome III were assessed using quantitative PCR analysis. We did not detect scc3-I358ins-6HA at either CAR whereas wild-type Scc3-6HA binding was easily detected (Fig. 3D). We repeated the ChIP experiment using an antibody against Mcd1 to determine whether cohesin trimer binding was also impaired by the scc3-I358ins-6HA mutation (Fig. 3E). As expected, Mcd1 was detected on the chromatin in the *SCC3-6HA scc3-6* strain. In contrast, Mcd1 binding was greatly reduced in the *scc3-I358ins-6HA scc3-6* strain. Therefore, the scc3-I358ins mutation dramatically reduces the level of cohesin binding to chromosomes.

There are two possible explanations for why the *scc3-I358ins* mutation compromises cohesin binding to chromosomes. Cohesin failed to load on chromosomes in S phase, or it dissociates prematurely from chromosomes in G2/M. To distinguish between these possibilities we examined the binding of scc3-I358ins-6HA to chromosomes by ChIP in early S phase by hydroxyurea (HU), instead of G2/M arrest (S2 Fig.). Cohesin was not bound to chromatin in HU arrested cells, suggesting that the *scc3-I358ins* affects initial loading in S phase rather than a failure to sustain binding in G2/M. Thus our phenotypic analyses of *scc3-I358ins* under physiological levels of expression corroborate and extend our studies of over-expression studies. The *scc3-I358ins* allele abrogates cohesin binding in S phase so consequently induces a major defect in cohesion establishment.

### Scc3 I358 region mediates binding of Scc3 to Mcd1

**Fig 4:**
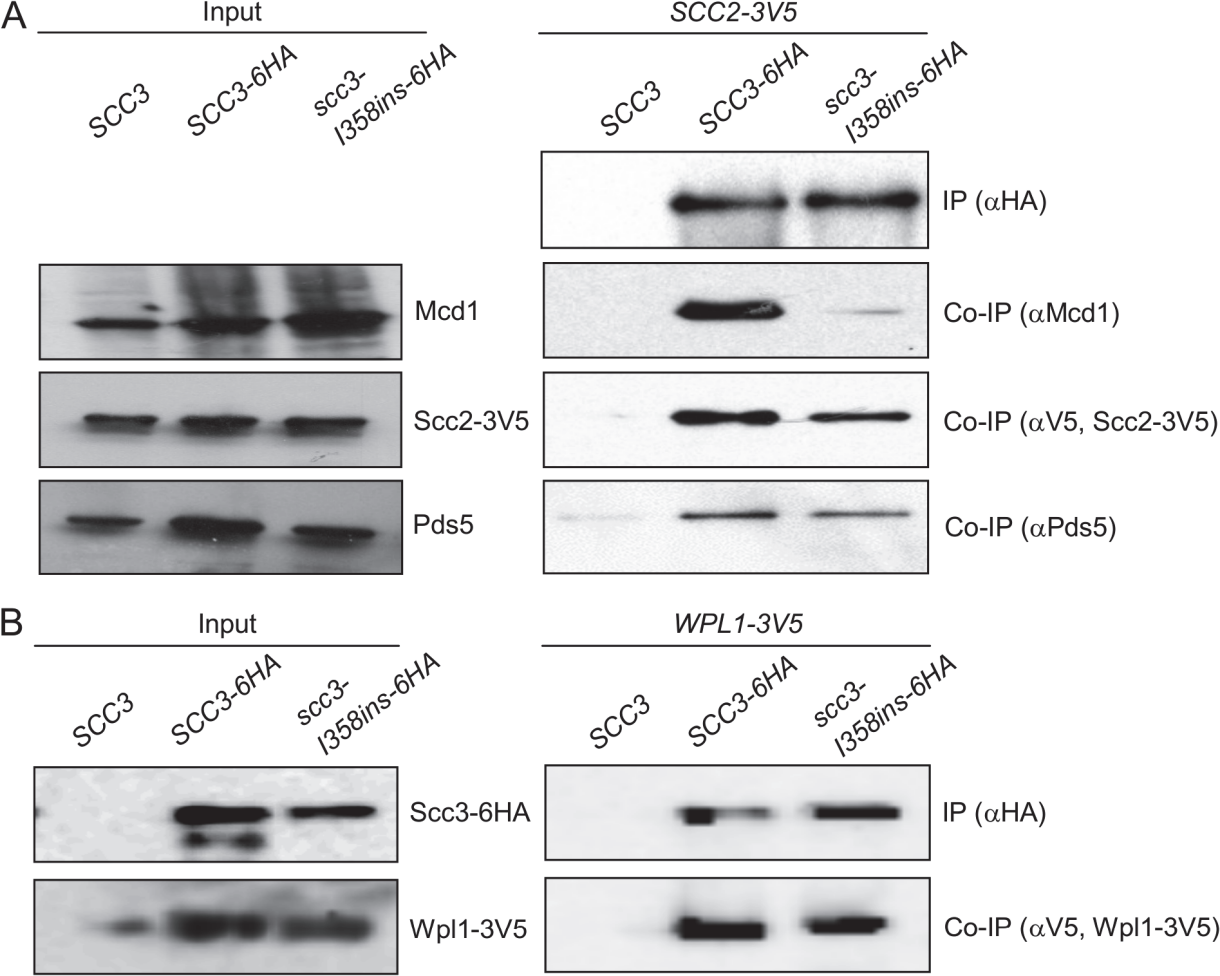
Co-immunoprecipitation of Scc3-I358Ins with core and accessory cohesin subunits. **A.** Analysis of scc3-I358ins-6HA binding to Mcd1, Scc2 and Pds5. Haploids JH5257 (*SCC2-3V5*), YIO92 (*SCC2-3V5 SCC3-6HA*) or YIO92R1 (*SCC2-3V5 scc3-I358ins-6HA*) cells were grown to mid-log phase in YPD media, lysed and subjected to immunoprecipitation against the HA tag (Scc3). Precipitated proteins were analyzed by Western blot using antibodies against HA (IP), Mcd1 (co-IP), V5 (co-IP) and Pds5 (co-IP). **B.** Analysis of scc3-I358ins-6HA binding to Wpl1. VG3333 (*WPL1-3V5*) YOG3007 (*WPL1-3V5 SCC3-6HA*) or YOG3008 (*WPL1-3V5 scc3-I358ins-6HA*) cells were analyzed as described in A. Precipitated proteins were analyzed by Western blot using antibodies against HA (IP) and V5 (co-IP).

The defect in *scc3-I358ins* allele likely arose from its inability to interact with the cohesin trimer via Mcd1 or one of the cohesin regulators known to bind Scc3, such as Scc2, Pds5 and Wpl1. To test this hypothesis, we examined the ability of Scc3-I358ins-6HA to co-immunoprecipitate with Mcd1, Scc2-3V5, Pds5 and Wpl1-3V5 using appropriate monoclonal or polyclonal antibodies (Fig. 4). We found that scc3-I358ins dramatically reduces the amount of Mcd1 that co-immunoprecipitates as compared to WT Scc3 (Fig. 4A). In contrast, scc3-I358ins and wild-type Scc3 bound Scc2-3V5, Pds5 and Wpl1-3V5 at similar levels (Fig. 4A and B). These analyses suggest that I358, and likely the entire RID A region, is required specifically for Scc3 to bind Mcd1. Furthermore, it corroborates previous *in vitro* studies that suggested that Scc3 can bind Scc2 and the Wpl1-Pds5 complex independently of its interaction with Mcd1 [24,26]. Finally, it strongly suggests that interaction of Scc3 with Mcd1 is necessary for cohesin loading and the establishment of sister chromatid cohesion.

### The Scc3-I358 region is required for a step in cohesin loading distinct from Scc2 binding to the cohesin trimer

**Fig 5:**
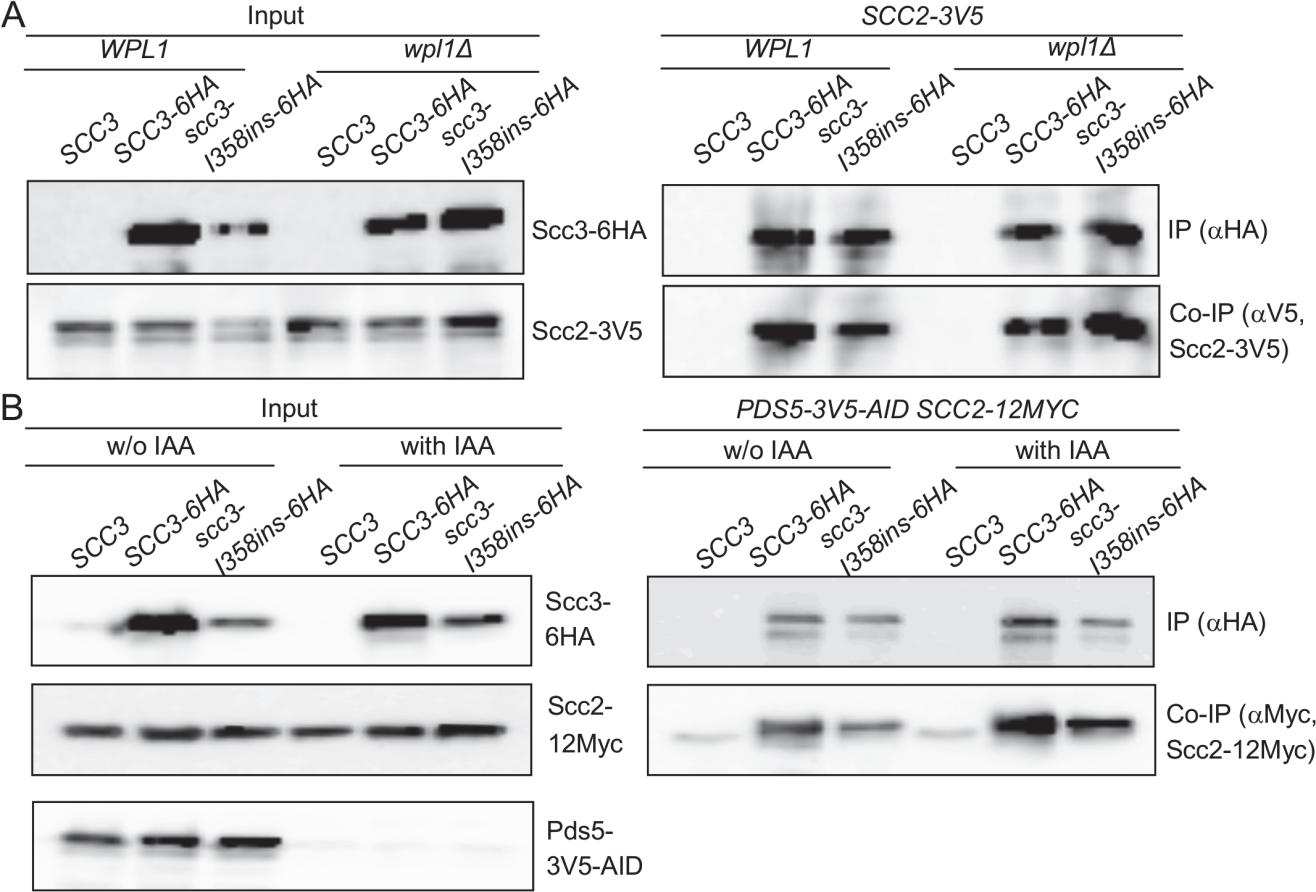
Impact of cohesin accessory factors on Scc2 binding to cohesin. **A.** Strains YOG3040 (*wpl1*Δ *SCC2-3V5*), YOG3002 (*wpl1*Δ *SCC2-3V5 SCC3-6HA*) or YOG3003 (*wpl1*Δ *SCC2-3V5 scc3-I358ins-6HA*) were grown to the mid-log phase in YEPD media. Cells were lysed and subjected to immunoprecipitation against the HA tag of Scc3. The co-precipitation of Scc2 was detected by anti V5 antibody. **B.** Strain YOG3050 (*PDS5-3V5-AID SCC2-12MYC*) YOG3044 (*PDS5-3V5-AID SCC2-12MYC SCC3-6HA*) and YOG3045 (*PDS5-3V5-AID SCC2-12MYC scc3-6HAI358ins-6HA*) were grown to the mid-log phase. Cells were divided into two flasks and 1 mM IAA was added to one of them for 60 minutes. The depletion of Pds5-3V5 was measured in the extract by Western blot with antibodies against V5. Cells were used for immunoprecipitation of Scc3 with anti-HA antibodies. The co-precipitation of Scc2 was detected by anti MYC antibody.

We sought to elucidate how the Scc3 interaction with Mcd1 promotes cohesin loading to chromosomes. We first asked whether the co-immunoprecipitation of the cohesin loader Scc2 with scc3-I358ins is dependent upon Wpl1 or Pds5. To address the contribution of *WPL1*, we deleted the gene from haploids strains bearing Scc2-12Myc and either Scc3-6HA or Scc3-I358ins-6HA. Cells were grown to mid-log phase, protein extracts prepared and Scc3-6HA or Scc3-I358ins-6HA immunoprecipitated using anti-HA antibodies. Scc2-3V5 efficiently co-immunoprecipitates with both Scc3-6HA and Scc3-I358ins-6HA (Fig. 5A). *PDS5* is an essential gene, so we utilized a conditional allele (*pds5-3V5-AID*) that can be rapidly and quantitatively destroyed by IAA addition [37]. Haploid Pds5-3V5-AID Scc2-12Myc strains bearing either Scc3-6HA or Scc3-I358ins-6HA were grown to mid-log phase, 1 mM IAA added and cells incubated for 1 hour to deplete Pds5. Protein extracts were prepared and Scc3-6HA or Scc3-I358ins-6HA immunoprecipitated using anti-HA antibodies. Western blot analysis showed that Pds5-3V5-AID was depleted to undetectable levels in comparison to untreated cells (Fig. 5A, left side). Pds5 depletion did not reduce Scc2-12Myc co-immunoprecipitation of either Scc3-6HA or Scc3-I358ins-6HA (Fig. 5A). Thus Scc3 and Scc2 co-immunoprecipitate even when Scc3 is unable to interact with Mcd1, Wpl1 or Pds5, suggesting that Scc3 directly interacts with Scc2.

**Fig 6:**
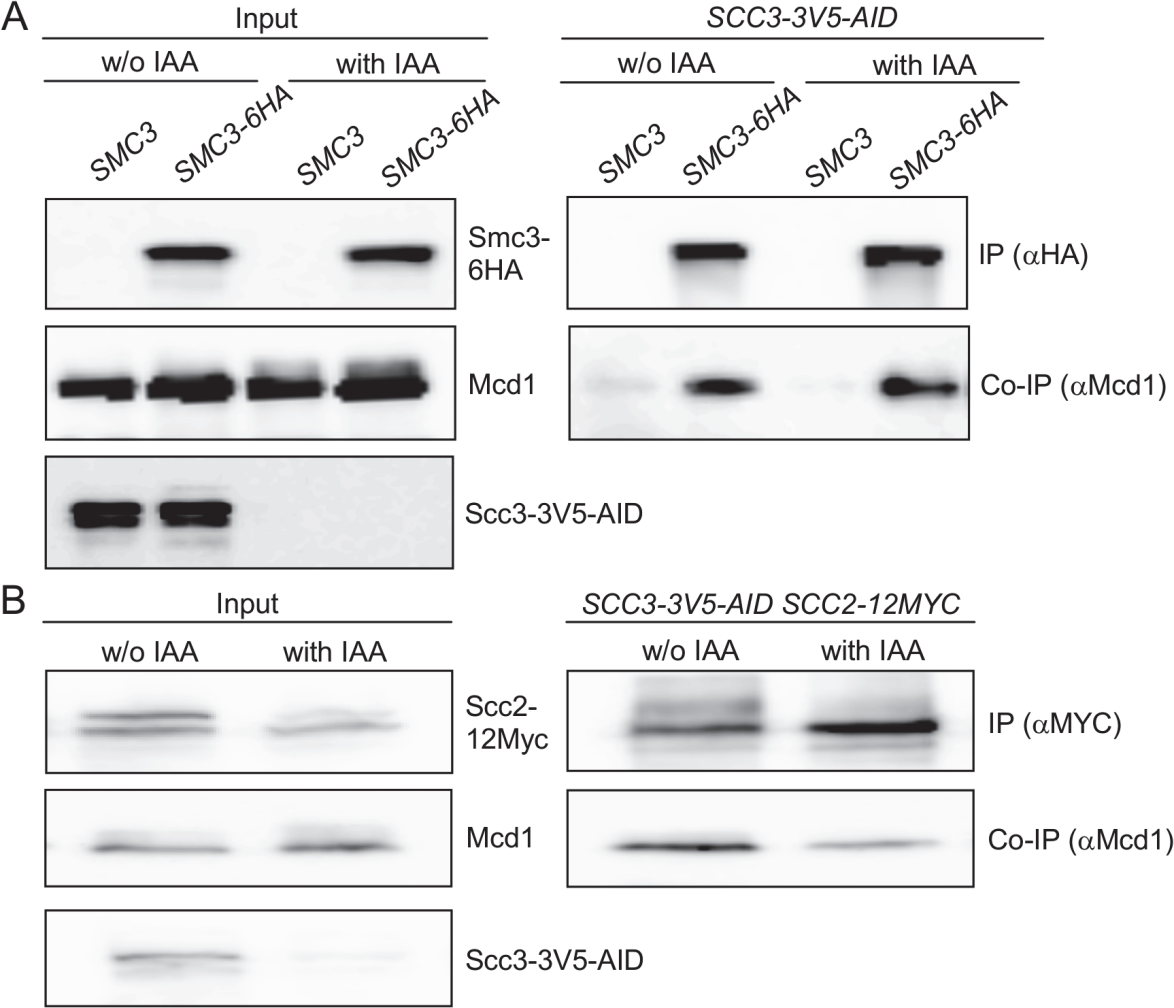
Scc3 does not affect the Smc3-Mcd1 interaction. **A.** Strain YOG3049 (*SCC3-AID-V5 SMC3-6HA*) was grown to the mid-log phase. Cells were divided into two flasks and 1 mM IAA was added to one of them for 60 minutes. The depletion of Scc3-3V5 was measured in the extract by Western blot with antibodies against V5. Un-depleted and depleted Scc3 cells were used for immunoprecipitation of Smc3-6HA with anti-HA antibodies. The co-precipitation of Mcd1 was detected by anti-Mcd1 antibody. **B**. Strain YOG3027 (*SCC3-AID-V5 SCC2-12MYC*) was grown to the mid-log phase. Cells were divided into two flasks and 1 mM IAA was added to one of them for 120 minutes. The depletion of Scc3-3V5 was measured in the extract by Western blot with antibodies against V5. Cells were used for immunoprecipitation of Scc2-12Myc with anti-MYC antibodies. The co-precipitation of Mcd1 was detected by anti-Mcd1 antibody.

These results raise the possibility that Scc3 binds to Scc2-Scc4, and then Scc3 interaction with Mcd1 recruits the loader to the cohesin trimer for interactions with other cohesin subunit. If so, scc3-I358ins, which is unable to bind Mcd1, could sequester Scc2-Scc4 away from the trimer and thereby prevent cohesin from binding chromosomes. To test this model we first asked whether Mcd1 can still interact with the Smc subunits in the absence of Scc3. Many experiments have shown that the trimer can form independently of Scc3 but only *in vitro*. To remove Scc3 in vivo, we generated an auxin-induced degron of Scc3, *SCC3-3V5-AID* [38,39]. *SCC3-3V5-AID* strains also contained an Smc3 allele that was tagged internally with a 6HA epitope. *SCC3-3V5-AID SMC3-6HA* cells were grown to mid-log phase then Scc3-3V5-AID was depleted by addition of 1 mM IAA and incubation for 1 hour. Western blot analysis showed that Scc3-3V5-AID was depleted to undetectable levels (Fig. 6A, left side). We immunoprecipitated Smc3-6HA and assayed its interaction with Mcd1. Smc3 co-immunoprecipitation with Mcd1 was not affected by depletion of Scc3 (Fig. 6A). We infer from this result that Scc3 is not essential for the formation of the Smc1-Smc3-Mcd1 trimer, corroborating the previous *in vitro* studies.

To test whether Scc2 could bind to the trimer independent of Scc3, we introduced the *SCC2-12MYC* allele into the *SCC3-3V5-AID* strain. IAA addition induced Scc3 depletion to undetectable levels (Fig. 6B, left side). After Scc3-3V5-AID depletion, Mcd1 was still able to bind to Scc2, albeit at slightly reduced levels compared to untreated cells (Fig. 6B). Given that Scc2 can bind the cohesin trimer independently of Scc3, the failure of scc3-I358ins-6HA containing cohesin to bind chromosomes likely reflects an Scc3 function required after the binding of Scc2 to the trimer.

### The N' terminal region of the stromalin conserved domain of Scc3 also mediates the interaction between Scc3 and Mcd1

**Fig 7:**
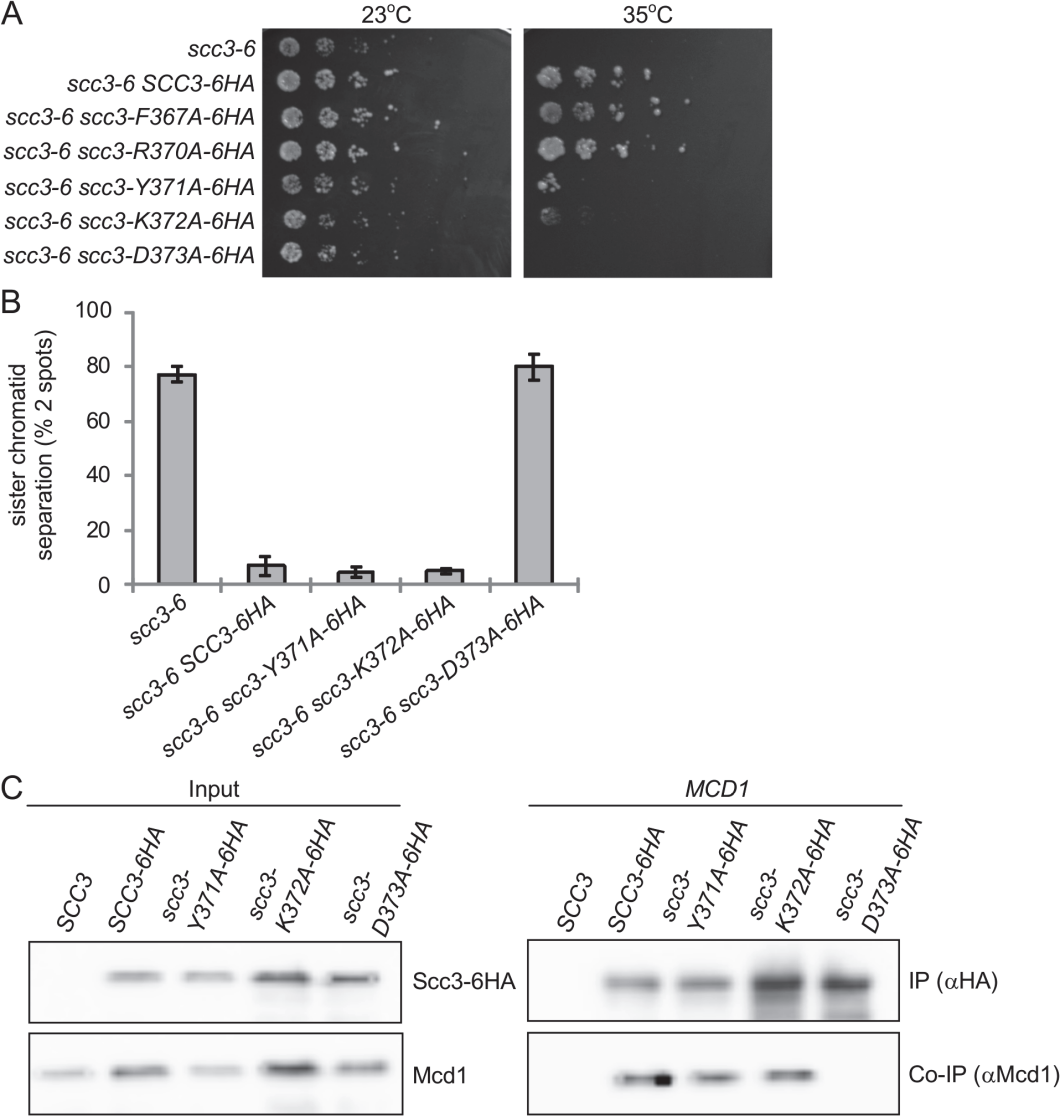
Analysis of alanine point mutations in Scc3’s SCD. **A.** Strain YIO81 (*scc3-6*), carrying an ectopic copy of *SCC3-6HA* (YOG3021), *scc3-F367A-6HA* (YOG3022), *scc3-R370A-6HA* (YOG3023), *scc3-*Y371A-6HA (YOG3024), *scc3-K372A-6HA* (YOG3025) or *scc3-D373A*-*6HA* (YOG3026) were grown to saturation in YPD media. Tenfold serial dilution of the strains were plated on YPD plates and grown at either permissive (23°C) or restrictive (35°C) temperature. **B**. Strains YIO81 (*scc3-6*), YOG3021 (*SCC3-6HA scc3-6*), YOG3024 (*scc3-Y371A-6HA scc3-6*), YOG3025 (*scc3-K372A-6HA scc3-6*) and YOG3026 (*scc3-D373A-6HA scc3-6*) were analyzed for sister chromatid cohesion using the GFP spot assay (n = 3). Cell cycle progression was determined by flow cytometry analysis (S4 Fig). **C**. Co-immunoprecipitation of Mcd1 with Scc3-6HA, scc3-Y371A-6HA, scc3-K372A-6HA or scc3-D373A-6HA. Precipitation of Scc3 was done by using an anti-HA antibody.

I358ins is located 9 amino acids upstream to the SCD (a.a. 367–457, Fig. 1D). Structural studies shows that the insertion region is located within an α-helix, while the downstream conserved region is within a short loop (S3 Fig.) [40]. Aiming to explore the functional relationship of the RID A region and the SCD, we individually mutated residues F367, R370, Y371, K372 and D373 to alanines. The mutant alleles also contained a C-terminal 6HA tag and were integrated into haploid strain YIO81 (*scc3-6*) at the *URA3* locus. Strains were tested for their ability to support cell viability by semi-quantitative serial dilutions plating at different temperatures (Fig. 7A).

As expected, the parent *scc3-6* strain is viable at 23°C but inviable at 35°C. The *scc3-6* cells bearing *scc3-6HA*, *scc3-F367A-6HA* or *scc3-R370A-6HA* alleles grew equally well at 35°C, suggesting that these residues do not impair Scc3 function. In contrast, strains bearing *scc3-Y371A-6HA*, *scc3-K372A-6HA* and *scc3-D373A-6HA* fail to complement the *scc3-6* mutant at 35°C. *scc3-Y371A-6HA* and *scc3-K372A-6HA* showed residual growth, while *scc3-D373A-6HA* was inviable. We then analyzed cohesion at the *LYS4* locus in these strains by arresting cells in G1, incubating for 1 hour at 35°C, and then allowing cell cycle progression at 35°C into G2/M phase arrest using nocodazole. The *scc3-D373A-6HA scc3-6* strain exhibited a defect in sister chromatid cohesion as severe as the *scc3-6* parent, as seen by the high percentage of cells with separated sister chromatids. Surprisingly, both the *scc3-Y371A-6HA* and *scc3-K372A-6HA* alleles complemented the cohesion defect of *scc3-6*, restoring cohesion to wild-type levels (Fig. 7B) even though both alleles failed to complement *scc3-6* for viability (Fig. 7A). Flow cytometry showed no difference in the cell cycle progression of either wild-type or mutants, excluding the possibility that the *scc3-Y371A-6HA* and *scc3-K372A-6HA* bearing strains failed to compete S phase (S4 Fig.). Finally, since the *scc3-Y371A* allele supports cohesion, we expected that Smc3 would be acetylated. Indeed, immunoprecipitation of Scc3-6HA or scc3-Y371A-6HA revealed equal amounts of both Smc3 co-immunoprecipitated and acetylated (S5 Fig.).

Due to the striking differences in the levels of sister chromatid cohesion between the *scc3-D373A-6HA* and the *scc3-Y371A-6HA* or *scc3-Y372A-6HA* alleles, we assessed whether they differed in their ability to assemble cohesin. For this purpose, asynchronous cultures of the scc3-6 cells bearing these alleles were grown to mid-log at permissive temperature (23°C). Protein extracts prepared and HA-tagged SCC3 subjected to co-immunoprecipitation to assess whether these mutant Scc3 proteins could bind Mcd1. The scc3-K372A-6HA and scc3-Y371A-6HA proteins co-precipitated with Mcd1, as well as the wild-type Scc3-6HA (Fig. 7C). In contrast, the scc3-D373A-6HA failed to co-precipitate with Mcd1 (Fig. 7C). Since scc3-I358ins-6HA also failed to bind Mcd1, these results suggest that the Scc3 region required for Mcd1 binding extends from the RID A region to the N-terminal part of the SCD.

### Scc3 is essential for chromosome condensation

**Fig 8:**
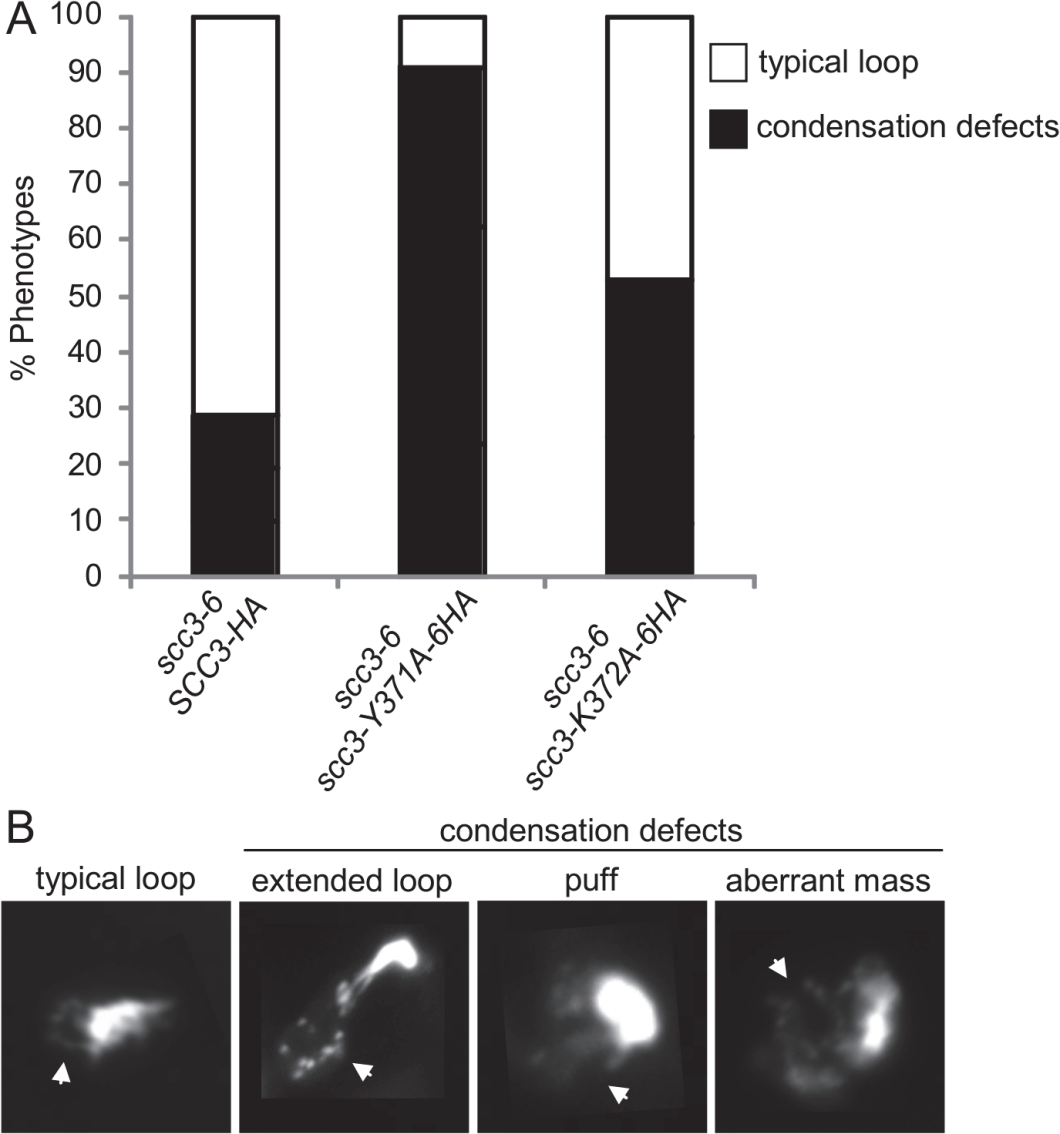
Analysis of chromosome condensation in Scc3 point mutants. **A.** Strains YOG3021 (*SCC3-6HA scc3-6*), YOG3024 (*scc3-Y371A-6HA scc3-6*) and YOG3025 (*scc3-K372A-6HA scc3-6*), were grown at permissive temperature (23°C) and arrested at G1 using α-factor. The cells were shifted to restrictive temperature (35.5°C) and re-arrested in the G2/M phase using nocodazole. Nuclei were spread and the bulk DNA was stained with DAPI. The morphology of the *rDNA*-loop of 100 nuclei of each strain was scored (n = 3). **B.** Representative photomicrographs of wild-type *rDNA* loops (left panel) and condensation defects at the *rDNA* locus. Arrow indicates the rDNA region.

The *scc3-K371A-6HA* and *scc3-Y372A-6HA* alleles have robust cohesion yet are inviable in a *scc3-6* background at the non-permissive temperature (Fig. 7A and B). Cohesin also mediates chromosome condensation, and this was proposed to be an essential cohesin function [35]. Therefore, we assessed whether these mutant alleles are competent for condensation. A haploid *scc3-6* parent strain alone or containing a second *SCC3* allele, either wild-type, *scc3-K372A-6HA* or *scc3-Y371A-6HA* were arrested in G1 at 23°C, shifted to 35°C for 1 hour to inactivate *scc3-6*, then released from G1 at 35°C and rearrested in G2/M using nocodazole. Chromosome spreads were prepared and stained with DAPI to enable condensation to be assessed at the *rDNA* locus as previously described [37]. About 70% of chromosomes with a typical *rDNA* loop were observed in cells carrying the wild-type allele (Fig. 8A and 8B). In contrast, about 5% of typical *rDNA* loops were detected with the *scc3-Y371A*-*6HA* allele (examples of typical *rDNA* loops and mutant phenotypes are shown in Fig. 8B). The condensation defect in the *scc3-K372A-6HA* mutant was milder. The results suggest that Scc3 plays an important function in chromosome condensation, independent of its role in sister chromatid cohesion establishment.

**Fig 9:**
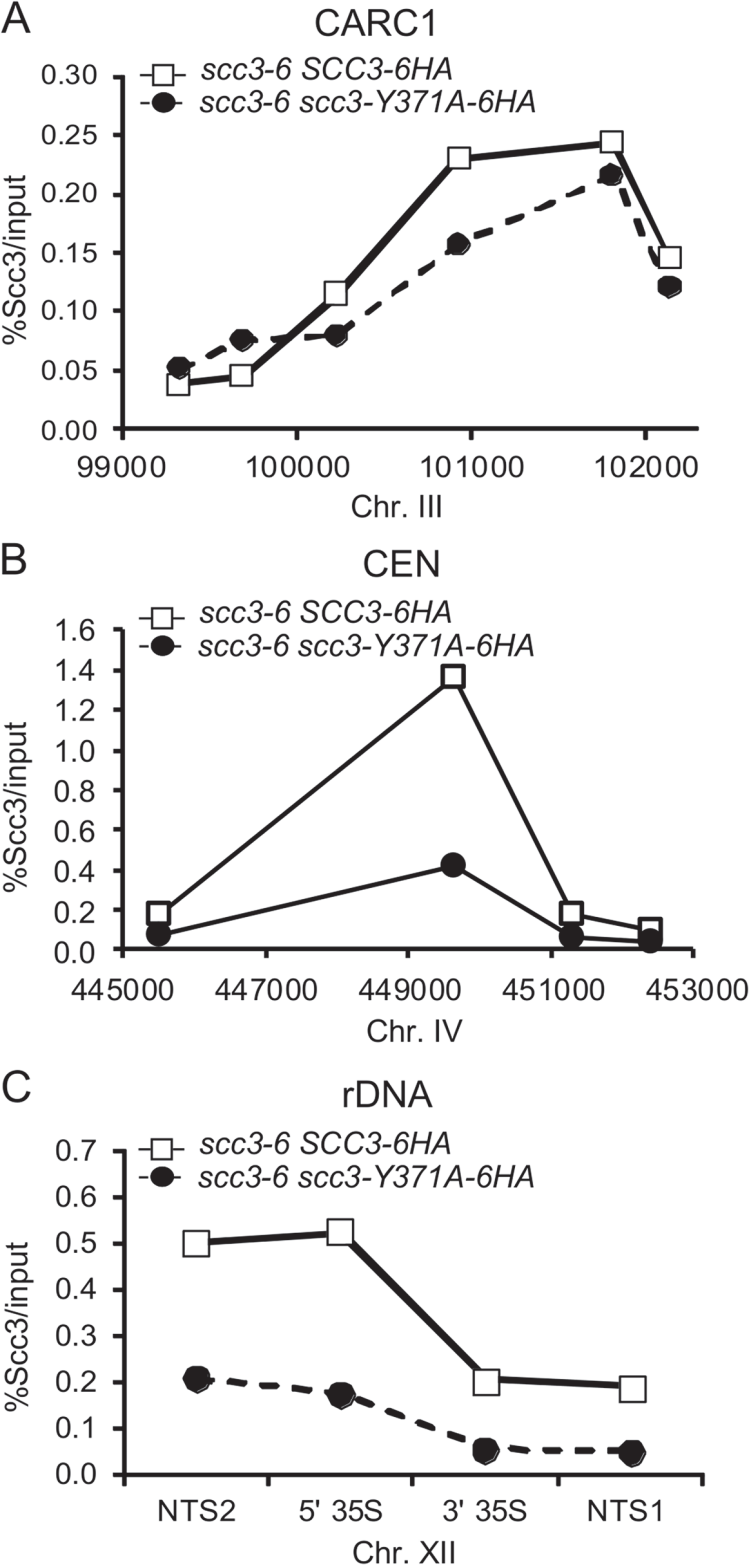
scc3 Y371 binding to the *rDNA* is reduced. Strains YOG3021 (*SCC3-6HA scc3-6*) and YOG3024 (*scc3-Y371A-6HA scc3-6*) were processed for chromatin immunoprecipitation analysis. Scc3 was immunoprecipitated with anti-HA antibodies and the precipitated DNA was analyzed by quantitative PCR for: **A.** chromosome III CARC1; **B.** chromosome IV centromere; **C.** chromosoXII *rDNA*. A representative PCR is shown (n = 3).

We next addressed what was the molecular defect in *scc3-Y371A* that allowed condensation but not cohesion? Given that this allele lay within a region that we showed was necessary for cohesin loading on chromosomes (between I358 and D373), it seemed likely that this allele also affected cohesin binding to chromosomes. Since *scc3-Y371A* was cohesion proficient, at least some cohesin must be loaded onto chromosomes. Therefore we anticipated that *scc3-Y371A* would reduce rather than abrogate cohesin binding to chromosomes. To test this possibility, we performed a ChIP assays by immunoprecipating the Scc3 Y371A-6HA protein and assaying its localization at CARs, centromeric DNA and the rDNA (Fig. 9). Cohesin binding on CARC1 on chromosome III was comparable to the wild type cells. Strikingly, about 2–3 fold reduction in cohesin binding to centromere of chromosome IV was found in scc3-Y371A cells as compared to wild type cells. Similar reduction was observed in the *rDNA* locus. Thus, the region encompassing RID A and SCD not only contains residues that controls global binding of cohesin to chromosomes, but also contains at least one residue (Y371) that controls binding to specific regions. The reduced loading of cohesin specifically at the centromere and rDNA in *scc3-Y371A* is consistent with a condensation defect as these regions appear to have unique condensation requirements as reflected by their robust recruitment of condensin [41,42].

## Discussion

Cohesin subunits and auxiliary proteins exhibit a complex pattern of physical and functional interactions. We performed a mutagenic screen of the Scc3 subunit of cohesin to help elucidate how it interacts with multiple binding partners to promote specific cohesin functions. A random insertion dominant screen identified an essential novel domain we termed RID A, which is adjacent to a previously identified conserved stromalin conserved domain (SCD). We characterized one RID A mutation, I358ins, and found it abrogates Scc3 association with Mcd1. Mutation of D373 in the neighboring SCD also abrogates Mcd1 binding. These two tightly clustered mutations likely define an Scc3 region critical for its interaction with Mcd1. We also showed that abrogation of this Scc3-Mcd1 interaction blocks cohesion establishment and localization of cohesin to chromosomes in S and M phases, consistent with a role for this Scc3-Mcd1 interface in cohesin loading on chromosomes.

While other genetic studies have shown that Scc3 is important for cohesin loading onto chromosomes, this function of Scc3 is elaborated significantly by our phenotypic analyses of the *scc3-I358ins* allele. The scc3-I358ins mutant protein, while defective for Mcd1 binding, still interacts with all the other known binding partners of Scc3 including cohesin regulators Pds5 and Wpl1, and the cohesin loader Scc2. Thus the RID A/SCD region of Scc3 is likely a specific docking site for Mcd1. Importantly, this Scc3-Mcd1 interaction is not required for Scc2 binding to the cohesin trimer since Scc2 continues to bind Mcd1 when the Scc3-Mcd1 interaction is abrogated by either by depleting Scc3 or the I358ins mutation. This *in vivo* Scc3 independence of the Scc2-cohesin trimer interaction fits with a recent *in vitro* study showing that Scc2 peptides bind to each subunit of the trimer in multiple places [26]. Several models posit that the cohesin loader alters the conformation of the cohesin trimer to mediate chromosome binding and cohesion. Our results suggest that loader binding to the trimer is not sufficient for loading. Rather Scc3 binding to Mcd1 is also needed for both cohesin binding to chromosomes and cohesion at a step distinct from Scc2 binding to the trimer.

How does the Scc3-Mcd1 interaction promote cohesin binding to chromosomes and cohesion? One possibility is that this interaction facilitates a loader function by a critical fine-tuning of its interaction with the Scc3-Pds5-Wpl1 trimer. When this interaction is missing in the scc3-I358ins mutant, Wpl1 immediately removes the trimer as soon as the loader puts it on chromosomes. Alternatively, the interaction of Scc3 with Mcd1 may facilitate loader function by altering loader interaction with the other sites on the cohesin trimer or with Scc3. Consistent with this model, the RID A-SCD region of Scc3 also binds an Scc2 peptide in vitro, so likely binds Scc2 [26]. Finally, it is possible that the Mcd1-Scc3 interaction plays a direct role in DNA binding and the tethering activity of cohesin. Models for cohesin function have focused on the sufficiency for the trimer for cohesin binding to chromosomes. Studies of the DNA binding for the bacterial SMC complexes, as well as the Rad50 Smc-like complex, reveal that nucleoprotein complex formation invokes very large conformational changes [43,44]. These changes leave room for Scc3 to play more complex roles in DNA binding beyond the putative topological entrapment of DNA by the trimer.

In an attempt to further define amino acids required for the Mcd1-Scc3 interaction, we discovered that mutating Y371 and K372 residues which are located between I358 and D373, induce cell inviability yet had no effect on sister chromatid cohesion or Scc3 binding to Mcd1 as assayed by co-immunoprecipitation. However, these mutations did cause a strong defect in mitotic chromosome condensation. The Y371 mutant reduced cohesin loading at the centromere and *rDNA*, but not at a centromere proximal arm site. This observation corroborates the idea that condensation is an essential function of cohesin in budding yeast [35]. Interestingly, yeast with reduced intracellular concentration of wild-type cohesin were viable, and exhibited a partial defect in *rDNA* condensation but not cohesion [45]. These cells like *scc3-Y371A* load less cohesin on the *rDNA* but unlike *scc3-Y371A* have normal levels of cohesin at the centromere. The difference in lethality may reflect differences in the level or type of condensation defect generated. Alternatively, the lethal condensation defect associated with cohesin may reflect a need for condensation at the centromere to ensure proper kinetochore function.

The defect in cohesin binding of Y371A and K372A suggests a unifying molecular mechanism to explain the apparently disparate phenotypes of the Y371 and K372 mutations compared to RID A and D373 mutations. This region mediates an interaction between Mcd1 and Scc3 that is required for loader function throughout the genome. Thus, mutations like I358ins and Y373A that block this interaction block loading. Residues within this region like Y371 and K372 modulate this global function further, either directly or via interaction with unknown partners to promote regional specific loading at the centromere and *rDNA*. Consequently, mutations like Y371A block this regional specific loading leading to defects in *rDNA* condensation. Most interestingly, it has been suggested that in human cells Scc3 homologs Sa1 and SA2 has distinct effect on cohesin binding. SA2 is involved in centromere cohesion, while SA1 mediates arm and telomere cohesion [46]. The differential cohesion binding induced by D373 and Y371 indicate that these distinct properties are conserved in yeast. Understanding how the interface between Scc3 and Mcd1 modulates loader function will provide important insights into the mechanism of cohesion and the distribution of cohesin on chromosomes.

## Materials and Methods

### Yeast strains and media

Yeast strains and plasmids used in this study are listed in S1 Table in the supporting information. Yeast strains were grown in SC–URA or YPD media, as described, supplemented with 2% glucose [47]. Media used for galactose inductions contained SC-URA supplemented with 3% glycerol, 2% lactic acid and 2% galactose.

### Cell synchronization

Cells were arrested in G1 phase by the addition of alpha-factor (1.5 X 10−^8^M final). To release cells from alpha-factor-induced G1 arrest, cells were washed 2 times with media containing pronase E (0.1 mg/ml; Sigma) and 2 times in media without pronase E. Exponentially growing cultures were arrested in G2/M using nocodazole (15 μg/ml final) in the indicated media. For early S arrest, hydroxyurea (200 mM; Sigma) was added.

### Random Insertion of Dominant negative (RID) screen

A library of mutant plasmids was prepared by using the Mutation Generation System Kit (F-701, Finnzyme/Thermo Scientific) on a *CEN3 URA3* plasmid bearing *SCC3* under control of the GAL promoter (*pGAL-SCC3*) according to the manufacturer’s instructions. Briefly, a transposon (Tn) reaction is conducted in vitro, and then the plasmid Tn insertion library is transformed into bacteria by using a selectable marker on the transposon. Enough single Tn insertions are generated in our library to yield one insertion every 2 bp. The Tn is excised by restriction digestion (a site at both ends of the Tn) and a new library is made by re-circularizing the plasmids, which leaves only a 15 bp insertion at the site of the initial Tn insertion (RID library). The RID library was transformed into haploid strain VG3135 (*scc3-6*). A ts strain grown at permissive temperature (23°C) was used to increase the sensitivity of the screen. Transformants were grown on SD-URA glycerol plates to select for the RID plasmid, but not to induce the SCC3-RID gene. Transformants were kept to a density of about 150 colonies per plate for ease of screening, and incubated at 23°C until the colonies formed. We then replica plated SC-URA plates containing either glucose (non-inducing) or galactose (inducing) and incubated at 23°C. Colonies that were inviable on galactose were re-tested to confirm this phenotype. Plasmids were isolated from galactose sensitive transformants and the location of the 15 bp insertion was determined by sequencing of the entire gene. To confirm linkage of inviability to candidate RID plasmid, hits were re-transformed into strain VG3135 and treated as described above to confirm that the *SCC3-RID* plasmid was responsible for toxicity on galactose media.

### Immunoprecipitation and Western blotting analysis

Cells were grown to mid-log phase, pelleted and washed with dH_2_O, and frozen in liquid nitrogen. Pellets were resuspended in 350 μl IPH150 or IPH50 buffer (50 mM Tris, pH 8.0, 150/50 mM NaCl, 5 mM EDTA, 0.5% NP-40, 1 mM DTT, protease inhibitors cocktail (Sigma)). Cells were lysed by adding glass beads (Sigma) to the re-suspended pellets followed by 4 working cycles of 1 minute in a bullet blender (Next Advance). The lysates were cleared by two centrifugations of 5 and 15 min at 15,000 g at 4°C. Immunoprecipitations were performed at 4°C adding the appropriate antibodies for 2 h. The antibodies were collected on protein A/G agarose (Santa Cruz) for 1 h and washed 3 times with IPH150 and resuspended in 35 μl Laemmli buffer. Standard procedures for sodium dodecyl sulfate–polyacrylamide gel electrophoresis and Western blotting were followed to transfer proteins from gels to a polyscreen PVDF membrane (Millipore). Membranes were blotted with the primary antibodies. Antibodies were detected using SuperSignal West Pico (Thermo) and LAS 4000 (GE). Antibodies used in this study: anti-HA (12CA5, Roche), anti-MYC (9E10, Roche), anti-V5 (Invitrogen/Millipore), Rabbit anti-Mcd1 (Rb555) and Rabbit anti-Pds5 (Rb558) antibodies were provided by Vincent Guacci.

### Auxin-induced depletion

Cells were grown to mid-log phase in YPD then split in half. 3-Indoleacetic acid (IAA, Sigma) was added to a final concentration of 1 mM to one half. The second culture was not treated with IAA but both halves were incubated an additional 2 h before being processed for immunoprecipitation as described above.

### Site Directed Mutagenesis

Site Directed Mutagenesis was performed on pIO97 (SCC3-6HA, URA3) by using QuikChange II XL Site-Directed Mutagenesis Kit (Agilent) following the manufacturer instructions. Primers used for the reactions are listed in supplementary S2 Table.

### Cohesion spot assay, chromosome spreads and Chromatin Immunoprecipitation (ChIP)

Cohesion at *LYS4* was assayed using the LacI-GFP/LacO array. Cells were treated as described in the text and processed to visualize GFP foci by microscopy as described previously in [33]. Each experiment was repeated three times and at least 300 cells were counted for each time-point in each experimental condition. Chromosome spreads were performed as described in [27]. ChIP was performed as described in [48]. Primers used for qPCR are listed in supplementary S4 Table

### Microscopy

Wide-field fluorescence images were obtained using the Zeiss AxioImager M2 fully motorized inverted microscope (100 X Plan-Apo, 1.4NA) fitted with an AxioCamHRm CCD High Resolution Camera.

## Supporting Information

S1 Fig Flow cytometry analysis. A representing analysis of cell cycle progression for the cohesion assay shown in Fig. 3B.
(PDF)

S2 Fig *scc3-I358ins* does not bind chromosome during S phase. Strains YIO91 (*SCC3-6HA scc3-6*) and YIO91R1 (*scc3-I358ins-6HA scc3-6*) were processed for chromatin immunoprecipitation analysis. HA tagged proteins were immunoprecipitated. Precipitated DNA was analyzed by quantitative PCR for chromosome IV centromere, as described (Material and methods). A representative experiment is shown (n = 3).
(PDF)

S3 Fig Structural analysis of Scc3 mutants. **A.** The tertiary structure of Scc3 (PDB 4UVK). Blue indicates the RID insertion region. The SCD is shown in red. **B.** Zoom in to the RID A region and the key residue Y371, K372 and D373.
(PDF)

S4 Fig Flow cytometry analysis. A representing analysis of the cell cycle progression in the cohesion and condensation experiments shown in Figs. 7B and 8A.
(PDF)

S5 Fig Smc3 is acetylated *in scc3 Y371A* background. Strains YIO081 (*scc3-6*), YOG3021 (*SCC3-6HA scc3-6*), YOG3024 (*scc3-Y371A-6HA scc3-6*) were grown to mid-log phase in YPD media, lysed and subjected to immunoprecipitation against the HA tag of Scc3. The acetylation state of the co-precipitated Smc3 was analyzed by Western blot using antibodies against K113 acetylated Smc3.
(PDF)

S1 Table Yeast strains. (DOCX)

S2 Table Primers used for site directed mutagenesis. (DOCX)

S3 Table The position of transposon insertions in the RID A region. (DOCX)

S4 Table Primers used for ChIP. (DOCX)
