# Supplementary figures and images for: A Conserved Domain in the Scc3 Subunit of Cohesin Mediates the Interaction with Both Mcd1 and the Cohesin Loader Complex
Source: PLoS Genet. 2015 Mar 6;11(3):e1005036. doi: 10.1371/journal.pgen.1005036 (PMC4352044; doi:10.1371/journal.pgen.1005036)

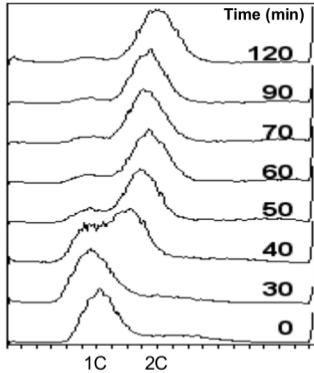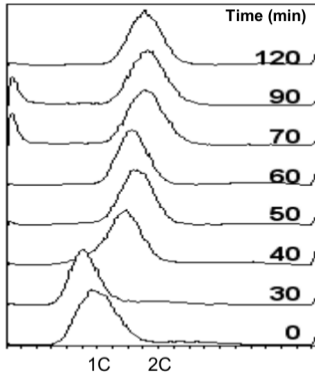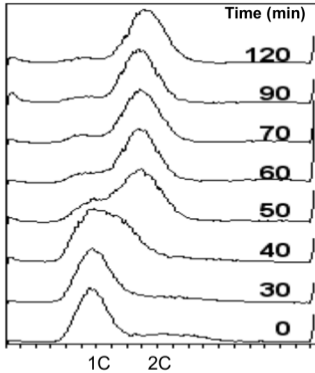

Supplement: S1 Fig — A representing analysis of cell cycle progression for the cohesion assay shown in Fig. 3B. (PDF) [file pgen.1005036.s001.pdf]

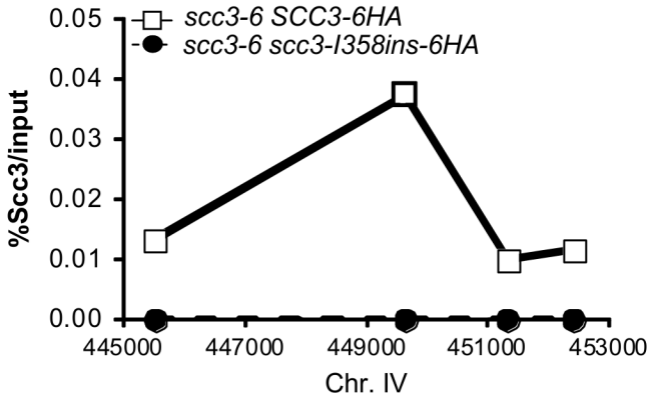

Supplement: S2 Fig — Strains YIO91 (SCC3-6HA scc3-6) and YIO91R1 (scc3-I358ins-6HA scc3-6) were processed for chromatin immunoprecipitation analysis. HA tagged proteins were immunoprecipitated. Precipitated DNA was analyzed by quantitative PCR for chromosome IV centromere, as described (Material and methods). A representative experiment is shown (n = 3). (PDF) [file pgen.1005036.s002.pdf]

**A**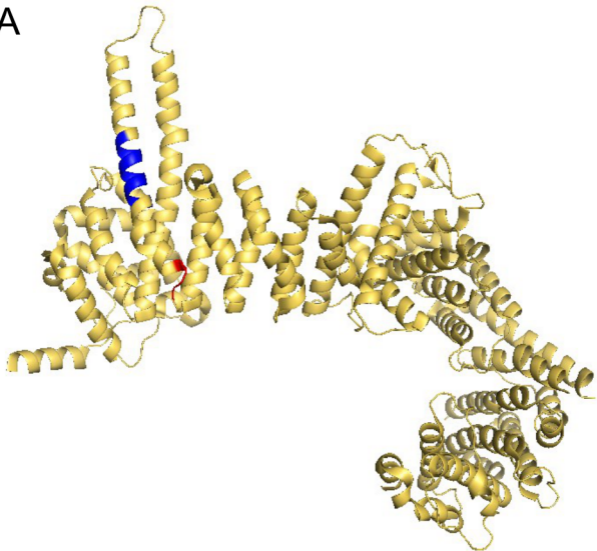**B**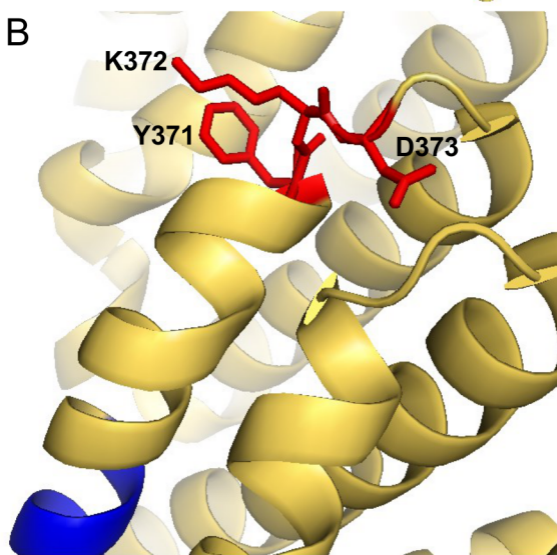

Supplement: S3 Fig — A. The tertiary structure of Scc3 (PDB 4UVK). Blue indicates the RID insertion region. The SCD is shown in red. B. Zoom in to the RID A region and the key residue Y371, K372 and D373. (PDF) [file pgen.1005036.s003.pdf]

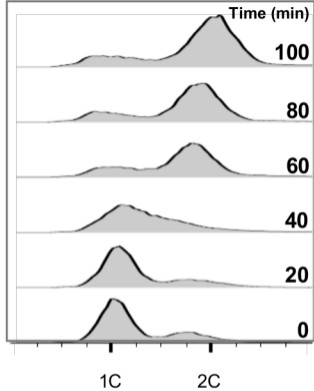

***Scc3-6***

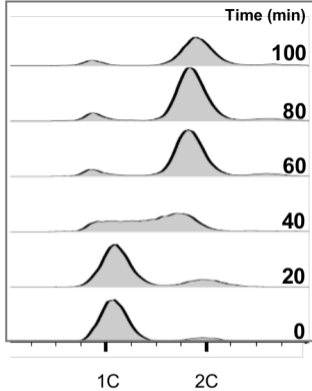

***Scc3-6 SCC3-6HA***

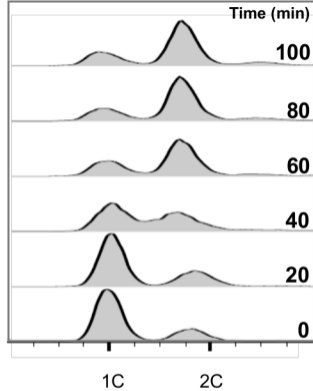

***Scc3-6 scc3-Y371A-6HA***

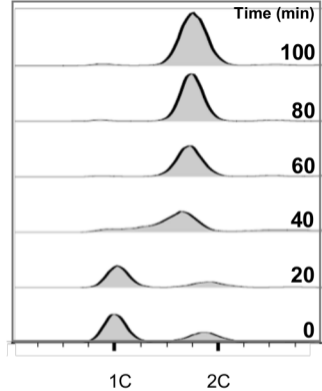

***Scc3-6 scc3-K372A-6HA***

Supplement: S4 Fig — A representing analysis of the cell cycle progression in the cohesion and condensation experiments shown in Figs. 7B and 8A. (PDF) [file pgen.1005036.s004.pdf]

Input

SCC3    SCC3-6HA    scc3-Y371A-6HA

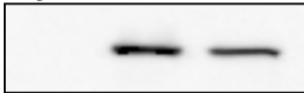

Scc3-6HA

*scc3-6*

SCC3    SCC3-6HA    scc3-Y371A-6HA

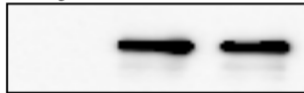

IP (αHA)

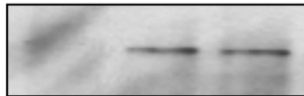

co-IP (αSmc3-Ac-K113)

Supplement: S5 Fig — Strains YIO081 (scc3-6), YOG3021 (SCC3-6HA scc3-6), YOG3024 (scc3-Y371A-6HA scc3-6) were grown to mid-log phase in YPD media, lysed and subjected to immunoprecipitation against the HA tag of Scc3. The acetylation state of the co-precipitated Smc3 was analyzed by Western blot using antibodies against K113 acetylated Smc3. (PDF) [file pgen.1005036.s005.pdf]
