# Supplementary material for: A Conserved Domain in the Scc3 Subunit of Cohesin Mediates the Interaction with Both Mcd1 and the Cohesin Loader Complex
Source: PLoS Genet. 2015 Mar 6;11(3):e1005036. doi: 10.1371/journal.pgen.1005036 (PMC4352044; doi:10.1371/journal.pgen.1005036)
Supplement: S1 Table — (DOCX) [file pgen.1005036.s006.docx]

**Supplementary Table S1. Yeast Strains**

| **Strain** | **Genotype** |
| --- | --- |
| VG3135 | *MATa scc3-6 trp1-1 leu2-3,112 ura3-52 his3-11,15 bar1 GAL+* |
| YIO81 | *MATa scc3-6 trp1-1 leu2-3,112 ura3-52 his3-11,15 bar1 GAL+his3::LACI-GFP-HIS3 lys4:LacO-NAT* |
| YIO81/pRS406 | *MATa scc3-6, trp1-1 leu2-3,112 ura3-52 his3-11,15 bar1 GAL+his3::LACI-GFP-HIS3 lys4:LacO-NAT+pRS406 (pGAL, URA3)* |
| YIO81/pIO88 | *MATa scc3-6, trp1-1 leu2-3,112 ura3-52 his3-11,15 bar1 GAL+his3::LACI-GFP-HIS3 lys4:LacO-NAT+pIO88 (pGAL-SCC3, URA3)* |
| YIO81/pIO88R1 | *MATa scc3-6 trp1-1 leu2-3,112 ura3-52 his3-11,15 bar1 GAL+ his3::LACI-GFP-HIS3 lys4:LacO-NAT+pIO88 (pGAL-scc3-I358ins URA3)* |
| YIO91 | *MATa scc3-6 trp1-1 leu2-3,112 his3-11,15 bar1 GAL+his3::LACI-GFP-HIS3 lys4:LacO-NATura3::SCC3-6HA-URA3* |
| YIO91R1 | *MATa scc3-6 trp1-1 leu2-3,112 his3-11,15 bar1 GAL+his3::LACI-GFP-HIS3 lys4:LacO-NATura3::scc3-I358ins-6HA-URA3* |
| JH5257 | *MATa trp1-1 leu2-3,112 his3-11,15 bar1 GAL+ his3::LACI-GFP-HIS3 lys4:LacO-NAT SCC2-3V5-G418* |
| YIO92 | *MATa trp1-1 leu2-3,112 his3-11,15 bar1 GAL+ pHIS3-GFP-LacI-HIS3:his3-11, Lys4:LacO:NAT, SCC2-3V5-G418, SCC3-6HA-URA3::ura3* |
| YIO92R1 | *MATa, trp1-1 leu2-3,112 his3-11,15 bar1 GAL+ pHIS3-GFP-LacI-HIS3:his3-11, Lys4:LacO:NAT, SCC2-3V5-G418, scc3-I358ins-6HA-URA3::ura3* |
| VG3333 | *MATa trp1-1 leu2-3,112 ura3-52 his3-11,15 bar1 WPL1-3V5-HIS3MX GAL+* |
| YOG3007 | *MATa trp1-1 leu2-3,112 his3-11,15 bar1 GAL+ WPL1-3V5-HIS3MXura3::SCC3-6HA-URA3* |
| YOG3008 | *MATa trp1-1 leu2-3,112 his3-11,15 bar1 GAL+, WPL1-3V5-HIS3MX ura3::scc3-I358ins-6HA-URA3* |
| YOG3040 | *MATa bar1 trp1-1 leu2-3,112 GAL+ wpl1∆::HPH lys4:LacO-NAT his3::LACI-GFP-HIS3 SCC2-3V5-G418* |
| YOG3002 | *MATa bar1 trp1-1 leu2-3,112 GAL+ wpl1∆::HPH lys4:LacO-NAT his3::LACI-GFP-HIS3 SCC2-3V5-G418 ura3::SCC3-6HA* |
| YOG3003 | *MATa bar1 trp1-1 leu2-3,112 GAL+ wpl1∆::HPH lys4:LacO-NAT his3::LACI-GFP-HIS3 SCC2-3V5-G418 ura3::scc3 I358ins-6HA-URA3* |
| YOG3021 | *MATa trp1-1 leu2-3,112 bar1 GAL+ scc3-6 his3::LACI-GFP-HIS3lys4:LacO:NAT ura3::SCC3-6HA-URA3* |
| YOG3022 | *MATa trp1-1 leu2-3,112 bar1 GAL+ scc3-6 his3::LACI-GFP-HIS3lys4:LacO:NAT ura3::scc3-F367A-6HA-URA3* |
| YOG3023 | *MATa trp1-1 leu2-3,112 bar1 GAL+ scc3-6 his3::LACI-GFP-HIS3lys4:LacO:NAT ura3::scc3-R370A-6HA-URA3* |
| YOG3024 | *MATa trp1-1 leu2-3,112 bar1 GAL+ scc3-6 his3::LACI-GFP-HIS3lys4:LacO:NAT ura3::scc3-Y371A-6HA-URA3* |
| YOG3025 | *MATa trp1-1 leu2-3,112 bar1 GAL+scc3-6 his3::LACI-GFP-HIS3lys4:LacO:NAT ura3::scc3-K372A-6HA-URA3* |
| YOG3026 | *MATa trp1-1 leu2-3,112 bar1 GAL+scc3-6 his3::LACI-GFP-HIS3 lys4:LacO:NAT ura3::scc3-D373A-6HA-URA3* |
| YOG3027 | *MATapep4∆::g418::TRP1 trp1-1 ura3-52 bar1 GAL+*  *his3::SCC2-12MYC-HIS3MX SCC3-3V5-AID2-G418leu2::TIR4-LEU2* |
| YOG3049 | *MATa SCC3-3V5-AID2-G418 TIR1-CaTRP1 LacO(DK) – NAT::lys4 pHIS3-GFPLacI-HIS3:his3-11,15 leu2-3,112 bar1 GAL+*  *ura3::SMC3-6HA:URA3* |
| YOG3050 | *MATa pep4∆::g418::TRP1 trp1-1,112 ura3-52,15 bar1 GAL+ PDS5-AID2:G418 TIR4:LEU2 his3::SCC2-12MYC: HIS3* |
| YOG3044 | *MATa pep4∆::g418::TRP1 trp1-1 bar1 GAL+ PDS5-AID2:G418 TIR4:LEU2 his3::SCC2-12MYC: HIS3*  *ura3::SCC3-6HA:URA3* |
| YOG3045 | *MATa pep4∆::g418::TRP1 trp1-1 bar1 GAL+ PDS5-AID2:G418 TIR4:LEU2 his3::SCC2-12MYC: HIS3*  *ura3::scc3-I358ins-6HA:URA3* |
