# Supplementary material for: A Conserved Domain in the Scc3 Subunit of Cohesin Mediates the Interaction with Both Mcd1 and the Cohesin Loader Complex
Source: PLoS Genet. 2015 Mar 6;11(3):e1005036. doi: 10.1371/journal.pgen.1005036 (PMC4352044; doi:10.1371/journal.pgen.1005036)
Supplement: S2 Table — (DOCX) [file pgen.1005036.s007.docx]

**Supplementary Table S2.Primers used for site directed mutagenesis**

| **Mutation** | **Forward primer** | **Reverse primer** |
| --- | --- | --- |
| F367A | catcgtcaaactatgtGCTgtgcataggtataag | cttatacctatgcacAGCacatagtttgacgatg |
| R370A | ctatgttttgtgcatGCGtataaggacgtgtc | gacacgtccttataCGCatgcacaaaacatag |
| Y371A | gttttgtgcataggGCTaaggacgtgtctg | cagacacgtccttAGCcctatgcacaaaac |
| K372A | gttttgtgcataggtatGCGgacgtgtctgatttg | caaatcagacacgtcCGCatacctatgcacaaaac |
| D373A | gtgcataggtataagGCCgtgtctgatttgattc | gaatcaaatcagacacGGCcttatacctatgcac |
