# Supplementary material for: A Conserved Domain in the Scc3 Subunit of Cohesin Mediates the Interaction with Both Mcd1 and the Cohesin Loader Complex
Source: PLoS Genet. 2015 Mar 6;11(3):e1005036. doi: 10.1371/journal.pgen.1005036 (PMC4352044; doi:10.1371/journal.pgen.1005036)
Supplement: S3 Table — (DOCX) [file pgen.1005036.s008.docx]

**Supplementary Table S3.The position of transposon insertions in the RID A region**

| Position of insertion | Number of clones |
| --- | --- |
| G350\|S351 | 2 |
| V354\|I355 | 1 |
| D356\|S357 | 2 |
| I358\|I359 | 3 |
