# Supplementary material for: A Conserved Domain in the Scc3 Subunit of Cohesin Mediates the Interaction with Both Mcd1 and the Cohesin Loader Complex
Source: PLoS Genet. 2015 Mar 6;11(3):e1005036. doi: 10.1371/journal.pgen.1005036 (PMC4352044; doi:10.1371/journal.pgen.1005036)
Supplement: S4 Table — (DOCX) [file pgen.1005036.s009.docx]

**Supplementary Table S4. Primers used for ChIP**

| Reverse | Forward | Location | |
| --- | --- | --- | --- |
| TGCTGTAGTCACCTCAGCAAG | AGCGGATCAATCCACAAAGC | 99327 | Chr. III  (arm) |
| AGCACTTTACTCGCTTGTGG | AAAGGTGCCCCAAGAAAAGG | 99690 |  |
| TGGGGGCTTCTCGATTTTTG | ATGCCAAGGCGGAAAGAATG | 100235 |  |
| GGCGTCAATGCTTTAGTTCTCC | ATGAGAAAGAGGGGTTCCTTCG | 100925 |  |
| CCAGCGATGAGATGCGAAAAG | ACTTTGGTTTTCCGGTGTGC | 101809 |  |
| ACGCGGAATTGAAACCACAG | ATGGTTCGGTTGGTGCTTAG | 102140 |  |
| AGCGGGCGGGTTATAAATAAC | TCGCTTTTCGCATCTCATCG | 101956 |  |
| TGATTGATTCACCTAGCCTT | GGTTGGGATCTAGGGATTAC | 445519 | Chr. IV (centromere) |
| TGATTATAAGCATGTGACCTTT | ACACGAGCCAGAAATAGTAAC | 449630 |  |
| ACAGCCCCCATTCTTG | GGAATACCGAGACCGTTAG | 451309 |  |
| ATACAAGCCAAGGACCG | CTTGGGTCTGTTGGGG | 452404 |  |
| GGGTGAACAATCCAACGCTT | CGTTCATAGCGACATTGCTT | NTS2 | Chr. XII (rDNA) |
| CTACACCCTCGTTTAGTTGC | TGTTAGTGCAGGAAAGCGGG | 3' 35S |  |
| GACTTACGTTTGCTACTCTC | GTATGTGGGACAGAATGTCG | 5' 35S |  |
| CACTAAGCCATTCAATCGGT | GCTTGCGTTGATTACGTCCC | NTS1 |  |
